# Imaging Findings of Paratesticular Adenomatoid Tumor

**DOI:** 10.1002/ccr3.71089

**Published:** 2025-11-12

**Authors:** Wenqin Liu, Zhifan Yuan, Limei Liang, Xiaoling Leng

**Affiliations:** ^1^ Department of Ultrasound The Tenth Affiliated Hospital of Southern Medical University, Dongguan People's Hospital Dongguan Guangdong China

**Keywords:** magnetic resonance imaging, paratesticular adenomatoid tumor, pathology, ultrasound

## Abstract

Paratesticular adenomatoid tumors are benign growths usually found near the epididymis. They often show no symptoms and appear as hyperechoic masses on ultrasound. Magnetic resonance imaging helps in difficult cases. Surgical removal is curative with minimal recurrence risk.

Paratesticular adenomatoid tumor, an uncommon benign neoplasm derived from mesothelial cells, predominantly arises in the epididymal vicinity of the testes. This entity comprises roughly 30% of all paratesticular tumors and approximately 60% of benign paratesticular lesions [1]. It can manifest across all age groups in males, with a predilection for sexually active young adults aged 20 to 50 years. Asymptomatic in nature, the detection of these tumors often occurs fortuitously, though a minority of patients may experience scrotal discomfort or pain secondary to mass enlargement. Here, we present a case of paratesticular adenomatoid tumor, its ultrasound, magnetic resonance imaging, and subsequent pathological confirmation.

A 47‐year‐old male patient presented to our hospital with a progressively enlarging left scrotal mass spanning 7 years. Physical examination revealed a drooping and swollen left scrotum harboring a palpable, round, smooth‐surfaced, painless mass that remained unchanged in size with positional manipulation. The transillumination test yielded negative results. High‐frequency ultrasound disclosed a heterogeneous, hyperechoic mass located at the caudal aspect of the left epididymis, demarcated by a clear boundary and interspersed with irregular internal echoes (Figure 1A). Upon probe compression, relative mobility was observed between the mass and the scrotal wall, accompanied by the discernment of short, rod‐like vascular signals both within and adjacent to the mass (Figure 1B). Magnetic resonance imaging provided further insights, revealing an enlarged left epididymis with irregular contours compared to the contralateral side. An abnormal signal intensity lesion was identified in the lower internal quadrant, exhibiting mildly prolonged T1 and T2 relaxation times. Moreover, focal, patchy areas with contrasting T1 shortening (Figure 2A) and T2 prolongation (Figure 2B) were noted. Enhanced magnetic resonance imaging scans demonstrated pronounced yet heterogeneous enhancement of the abnormal signal nodules within the lower inner aspect of the left epididymis and scrotum (Figure 2C). The patient underwent left epididymal mass resection, and pathological assessment confirmed the diagnosis of adenomatoid tumor with localized necrosis (Figure 3). Immunohistochemical profiling revealed positive staining for CK, Vim, WT‐1, EMA, CK5/6, CK7, CR, MC, and CD31/CD34, whereas CEA, CK20, Prohibitin a, Oct‐4, and SALL4 were negative. The Ki‐67 index was approximately 3%.

**FIGURE 1:**
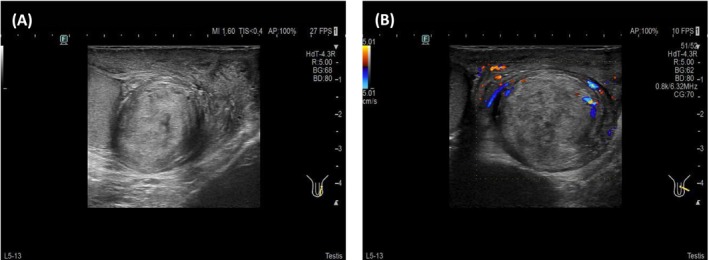
Ultrasound of paratesticular adenomatoid tumor. (A) Grayscale ultrasound showed an irregular, well‐defined, heterogeneous, and hyperechoic mass located at the caudal aspect of the left epididymis. (B) Color Doppler flow imaging showed short, rod‐like vascular signals both within and adjacent to the mass.

**FIGURE 2:**
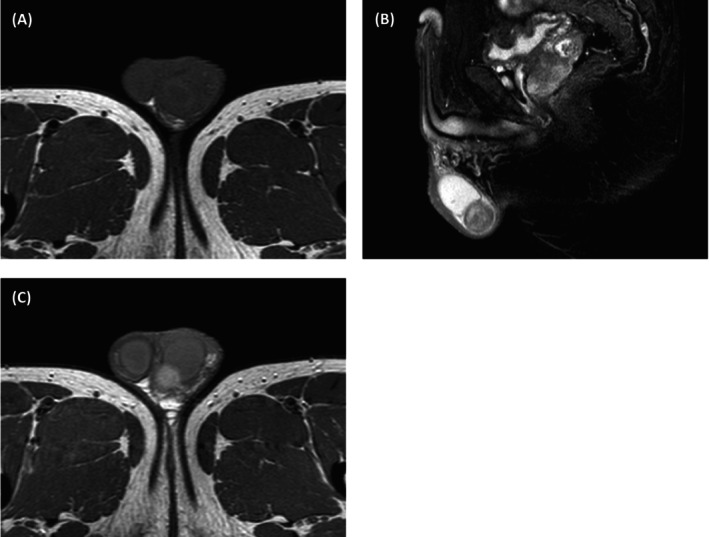
Magnetic resonance imaging of paratesticular adenomatoid tumor. (A) T1 weighted image showed the mass was slightly high signal with a patchy low signal. (B) T2 weighted image showed that the mass was high, with a patchy low signal. (C) Enhanced magnetic resonance imaging scans showed the mass was a heterogeneous enhancement.

**FIGURE 3:**
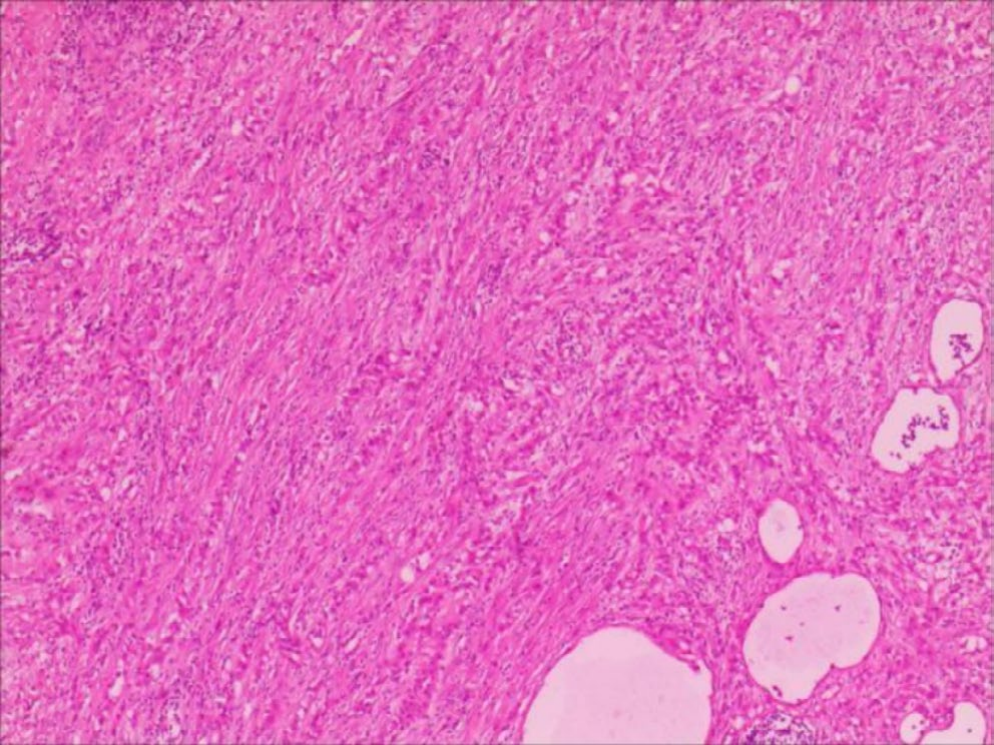
Pathological examination of paratesticular adenomatoid tumor. Hematoxylin and Eosin staining showed the cells with pale cytoplasm were glandular, tubular, and cord‐like invasive growth.

Ultrasound serves as the initial modality of choice for diagnosing testicular adenomatoid tumors. Tumors in the epididymal tail commonly manifest as hyperechoic nodules, whereas those in the head may exhibit varied echogenicity. Tumors are typically located between the epididymal head and the testicular upper pole [2]. In cases where ultrasound diagnosis is challenging or local infiltration is suspected, magnetic resonance imaging offers a more exhaustive assessment, enabling precise localization, sizing, morphological delineation, and assessment of lesion‐tissue relationships, thereby facilitating differential diagnosis from other scrotal pathologies [3]. However, magnetic resonance imaging is usually unnecessary and may be cost‐prohibitive in most cases. The mainstay of paratesticular adenomatoid tumor management involves local surgical excision without the necessity for testicular resection, ensuring complete tumor removal to prevent recurrence. Surgical intervention typically results in a complete cure, with low rates of postoperative recurrence and malignancy.

## Author Contributions

**Wenqin Liu:** data curation, writing – original draft. **Zhifan Yuan:** data curation, formal analysis. **Limei Liang:** data curation, formal analysis. **Xiaoling Leng:** supervision, writing – review and editing.

## Disclosure

Transparency Statement: We can confirm that this manuscript is an honest, accurate, and transparent account of the case being reported and that no important aspects have been omitted.

## Consent

Written informed consent was obtained from the patient to publish this manuscript by the journal's patient consent policy.

## Conflicts of Interest

The authors declare no conflicts of interest.

## Data Availability

The data used to support the findings of this manuscript are available from the corresponding author upon request.
